# Hypoplastic Celiac Trunk With Aberrant Hepatic and Splenic Arteries: A Case Report

**DOI:** 10.7759/cureus.87201

**Published:** 2025-07-02

**Authors:** Bandar M Abuageelah, Mona H Alfaifi, Yousef M Alyami, Hamzah M Alyami, Asrar G Alqahtani, Ahmed E Omara, Mohammed H Alshehri

**Affiliations:** 1 General Medicine, Batterjee Medical College, Aseer, SAU; 2 General Surgery, Saudi German Hospital Aseer, Aseer, SAU; 3 Bariatric Surgery and Endocrinology, Saudi German Hospital Aseer, Aseer, SAU

**Keywords:** aberrant hepatic artery, aberrant splenic artery, acute abdomen, ct angiography, hypoplastic celiac trunk, vascular anomaly

## Abstract

Vascular anomalies of the celiac trunk and its branches are rare but carry significant diagnostic and surgical implications. We report a case of a 24-year-old male presenting with acute abdominal pain and vomiting. Contrast-enhanced CT revealed a hypoplastic celiac trunk with aberrant hepatic and splenic arteries arising from the superior mesenteric artery (SMA), a configuration distinct from the more commonly described replaced right hepatic artery. Associated findings included dilated duodenum and jejunum with wall thickening and mesenteric inflammation, likely reflecting altered mesenteric hemodynamics. The patient was managed conservatively for an acute abdomen with suspected transient vascular congestion. This rare vascular variant highlights the importance of CT angiography in identifying atypical anatomy, which is crucial for preventing misdiagnosis and guiding safe surgical or interventional procedures.

## Introduction

The celiac trunk is a critical branch of the abdominal aorta, typically supplying the foregut, which includes the liver, stomach, spleen, and pancreas, via its three main branches: the left gastric, common hepatic, and splenic arteries. However, anatomical variations in this vasculature are not uncommon, with reported incidences ranging from 10% to 40% in cadaveric and angiographic studies [1-3]. Among these variations, a hypoplastic celiac trunk, characterized by an unusually small or underdeveloped trunk, paired with aberrant hepatic and splenic arteries, has a reported prevalence of less than 1-2% in angiographic studies and presents a unique clinical and surgical challenge [4-5].

A hypoplastic celiac trunk may fail to provide sufficient blood flow, leading to compensatory collateral circulation or atypical branching patterns [5]. In some cases, the hepatic and splenic arteries arise separately from the aorta or superior mesenteric artery (SMA), bypassing the celiac trunk entirely, a configuration that has significant implications for hepatobiliary and pancreatic surgeries, as well as interventional radiology procedures [6].

This variant arises embryologically from the persistence of the primitive ventral splanchnic artery associated with the SMA and regression of the celiac segment, disrupting the typical foregut arterial supply [6-8]. While many individuals remain asymptomatic, recognition of these variations is crucial to avoid iatrogenic injuries during procedures like liver transplantation, pancreatic resection, or transarterial chemoembolization (TACE) [9,10].

Understanding these variations is critical for accurate diagnostic imaging, surgical planning, and avoidance of iatrogenic complications. This study explores the embryological basis, clinical implications, and diagnostic considerations of a hypoplastic celiac trunk with aberrant hepatic and splenic arterial anatomy.

## Case presentation

A 24-year-old single male presented to the emergency department with severe, acute-onset abdominal pain that had progressed over the past four days. The pain was associated with multiple episodes of vomiting. On physical examination, the patient demonstrated limited abdominal movement with respiration and marked tenderness localized to the right iliac fossa and pelvic region. Initial blood tests revealed leukocytosis with a white blood cell (WBC) count of 20,000/mm³ (reference range: 4,000-11,000/mm³). Arterial blood gas (ABG) analysis was normal, with no evidence of metabolic acidosis.

Imaging studies

**Figure 1:**
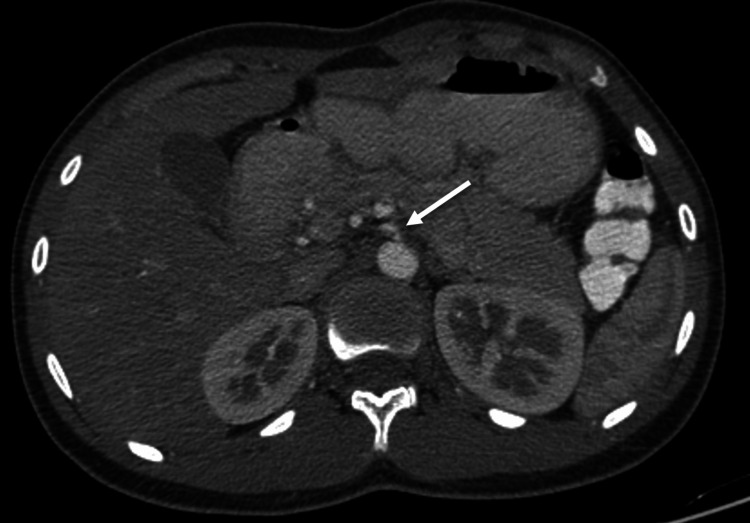
Axial contrast-enhanced CT at the T12–L1 level shows a hypoplastic celiac trunk (arrow) arising from the aorta, demonstrating reduced caliber in cross-sectional view.

**Figure 2:**
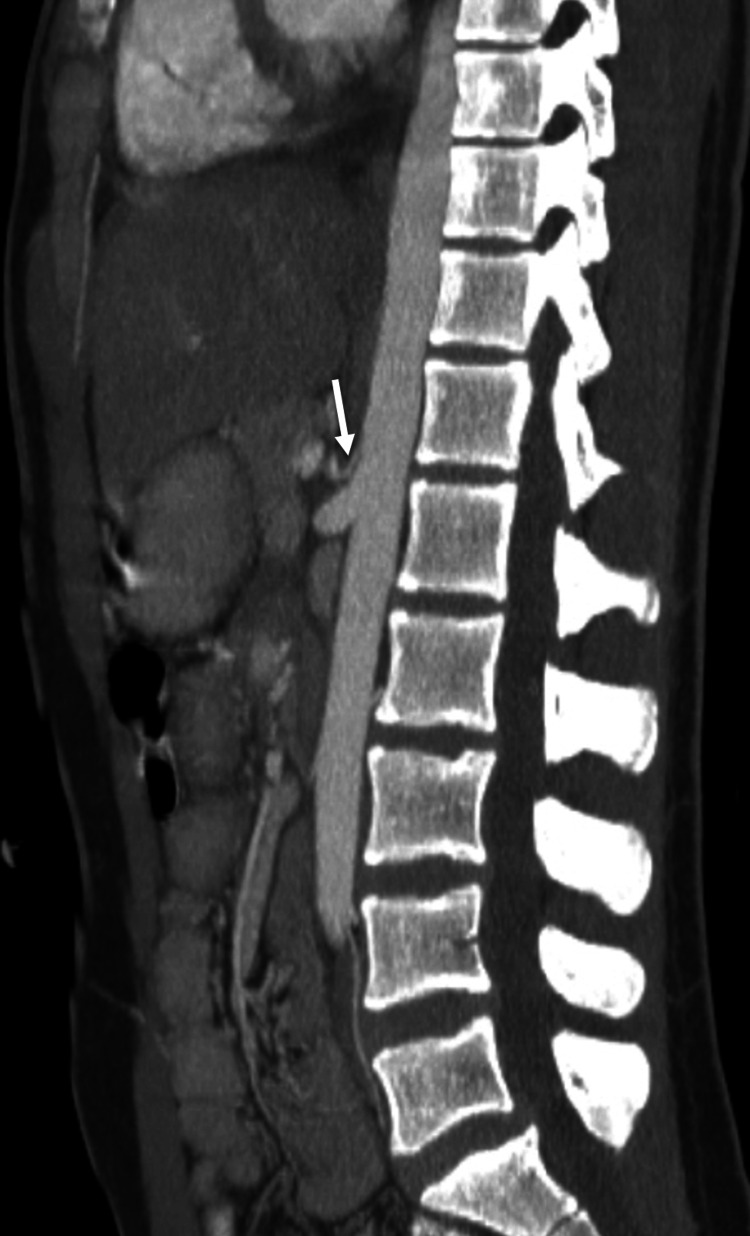
Sagittal MIP CT image highlighting the anteroposterior orientation and attenuated celiac trunk, demonstrating its limited contribution to visceral arterial supply.

**Figure 3:**
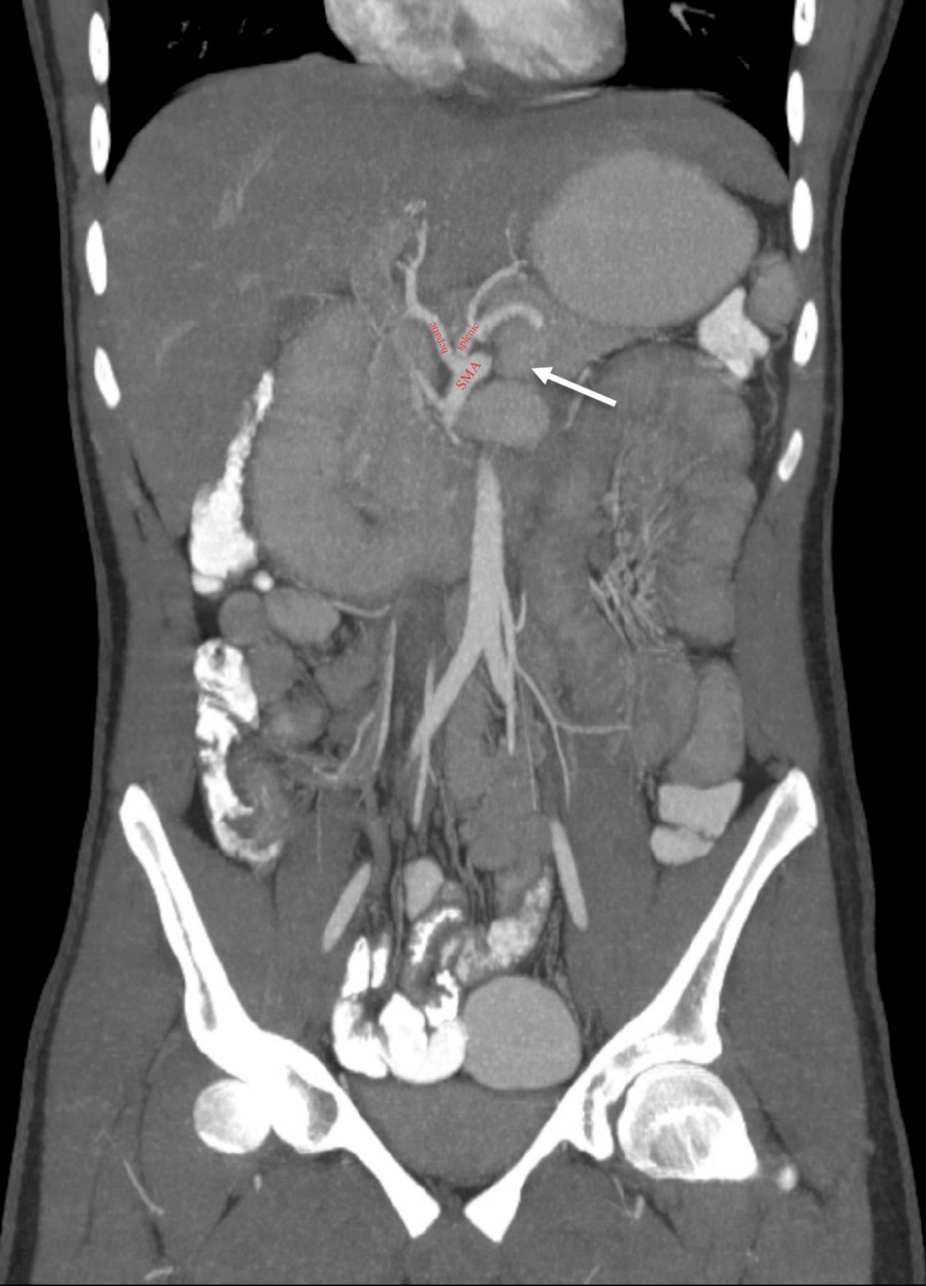
Coronal MIP contrast-enhanced CT shows aberrant hepatic and splenic arteries arising from the SMA (labels).

An erect abdominal X-ray showed multiple air-fluid levels, suggestive of intestinal obstruction. Further evaluation with contrast-enhanced computed tomography (CT) of the abdomen and pelvis (oral and intravenous contrast) revealed a hypoplastic celiac trunk. Notably, both the hepatic and splenic arteries were found to originate aberrantly from the SMA. By contrast, the left gastric artery arose normally from the celiac trunk. In addition, the duodenum and the proximal jejunum were dilated with thickened walls, and there was evidence of inflammatory changes at the root of the mesentery, as presented in Figures 1-3.

Given the absence of metabolic acidosis, peritoneal signs, or evidence of acute mesenteric ischemia, the vascular anomaly was initially considered a potential, but not definitive, contributor to the obstructive symptoms, possibly through altered mesenteric perfusion. Key differentials included early adhesive small bowel obstruction, subacute appendicitis with reactive ileus or mesenteric lymphadenitis. The patient was managed conservatively with intravenous fluids, analgesia, and close clinical monitoring. Gradual resolution of symptoms over 48 hours supported a transient and non-surgical process. The patient was discharged in stable condition with scheduled outpatient follow-up, including repeat imaging to monitor for recurrence or evolving mesenteric pathology related to the vascular variant. No symptoms were reported at the four-week review.

## Discussion

This case of a 24-year-old male presenting with severe, acute-onset abdominal pain, progressively worsening over four days, and signs of intestinal obstruction reveals several clinically significant vascular anatomical variations that warrant careful consideration. The imaging findings of a hypoplastic celiac trunk with both hepatic and splenic arteries originating aberrantly from the SMA represent a rare vascular configuration that has important implications for diagnosis, surgical planning, and patient management. Embryologically, this variant likely results from abnormal development of the ventral splanchnic arteries between the 4th and 8th weeks of gestation, where excessive regression, rather than incomplete fusion, of the primitive vascular network leads to the persistence of atypical arterial pathways [11]. The hypoplastic celiac trunk in this case indicates either failure of normal development or subsequent involution, with the SMA compensating by providing collateral circulation to both hepatic and splenic vascular territories [12]. This anatomical arrangement has been documented in approximately 1-2% of cadaveric studies, making it a rare but clinically important variant [1]. This anatomical configuration differs from more common variants such as a replaced right hepatic artery from the SMA or a splenic artery from the aorta, in that both major branches of the celiac trunk (hepatic and splenic arteries) arise aberrantly from the SMA, while the celiac axis itself is markedly hypoplastic and supplies only the left gastric artery. This complete dual origin from the SMA with no dominant celiac trunk contribution is exceptionally rare and functionally equivalent to celiac trunk agenesis.

The clinical presentation in this case reveals several clinical considerations. First, the mesenteric inflammatory changes and proximal small bowel dilation observed on imaging may represent either a primary intestinal pathology or secondary changes related to altered mesenteric hemodynamics. While the patient's leukocytosis and obstructive symptoms initially proposed mechanical obstruction or acute appendicitis, the vascular anomalies raise the possibility of chronic mesenteric congestion contributing to the clinical picture. Similar cases in the literature have described how aberrant hepatic arterial anatomy can predispose to biliary ischemia or complicate hepatobiliary surgeries [13]. The absence of metabolic acidosis on arterial blood gas analysis suggests that acute mesenteric ischemia was not yet present, though the risk remains elevated in such vascular configurations [14]. However, alternative causes, such as early adhesive small bowel obstruction or mesenteric lymphadenitis, were also considered, given the imaging findings and leukocytosis. Although the rapid resolution with supportive care favored a transient vascular or inflammatory origin, these alternative causes were also considered.

From a surgical perspective, this case underlines the critical importance of preoperative vascular mapping. The aberrant arterial anatomy presents substantial risks during abdominal procedures, particularly hepatobiliary or pancreatic surgeries, where inadvertent injury to these variant vessels could lead to significant hemorrhage or organ ischemia [15]. Several case reports have documented iatrogenic injuries during laparoscopic cholecystectomy when variant hepatic arteries were not identified preoperatively [16]. The current case highlighted the value of contrast-enhanced CT angiography as the gold standard for delineating such anatomical variations before intervention [17]. Furthermore, the hypoplastic celiac trunk may have implications for endovascular procedures, as the altered vascular anatomy could affect catheterization routes and embolization strategies [18]. This case emphasizes the importance of preoperative vascular mapping, as dual aberrant origins of hepatic and splenic arteries from the SMA carry a significant risk during abdominal surgery and may complicate endovascular access. CT angiography remains the gold standard for identifying such variants [17], enabling tailored surgical planning and alternative catheterization strategies such as SMA-based or radial access.

When compared with similar cases in the literature, several distinctive features emerge. While replaced right hepatic arteries originating from the SMA are relatively common (occurring in 11-15% of individuals), the absence of dominant celiac trunk contribution, with a hypoplastic trunk supplying only the left gastric artery, combined with dual aberrant origins from the SMA, is exceptionally rare [19]. The clinical presentation in this case differs from more commonly reported median arcuate ligament syndrome, where extrinsic compression of the celiac trunk typically causes postprandial pain rather than acute obstructive symptoms [14]. The mesenteric inflammatory changes observed here may represent a unique pathophysiological consequence of chronic vascular rearrangements, indicating that such anatomical variants may predispose to subclinical mesenteric congestion that manifests acutely under stress [20], although no direct evidence confirms causality in this case. Ugurel et al. reported hepatic artery anomalies in 4.5% of cases, but this case's dual SMA origin with celiac hypoplasia is far less common [5]. Winston et al. described an SMA-originating hepatic artery causing mesenteric ischemia, supporting the relevance of angiographic mapping in atypical abdominal pain [9]. Unlike Michels’ classification of replaced hepatic arteries, our case reflects not replacement but a complete shift in arterial supply due to celiac hypoplasia [6].

The management of this case required careful consideration of both the acute presentation and the underlying anatomical variation. Conservative management with close monitoring was initially appropriate given the absence of ischemic markers, though the potential for progression to complete obstruction or ischemia necessitated surgical readiness [21]. Long-term follow-up would be advisable to monitor for potential complications such as chronic mesenteric insufficiency or vascular steal phenomena between the hepatic and splenic circulations [22]. This case contributes to the growing body of literature emphasizing that vascular anatomical variations are not merely academic curiosities but can have real clinical consequences that impact presentation, diagnosis, and treatment outcomes. Given this unusual anatomy, we recommend outpatient follow-up to monitor for signs of chronic mesenteric insufficiency and, if clinically indicated, surveillance imaging such as CT angiography to assess for evolving perfusion compromise.

## Conclusions

This case underscores the importance of recognizing vascular anomalies in patients presenting with acute abdominal pain, particularly rare variants such as dual aberrant origins of the hepatic and splenic arteries from the SMA with a hypoplastic celiac trunk. While no definitive causal relationship was established between the vascular anomaly and the patient's obstructive symptoms, the anatomical configuration may predispose to altered mesenteric hemodynamics or complicate diagnosis and management. Contrast-enhanced CT remains the gold standard for accurate vascular mapping and procedural planning. Further observational and anatomical prevalence studies are warranted to better characterize the incidence, clinical significance, and potential mesenteric consequences of hypoplastic celiac trunk variants.
